# Effects of *Erxian *decoction, a Chinese medicinal formulation, on serum lipid profile in a rat model of menopause

**DOI:** 10.1186/1749-8546-6-40

**Published:** 2011-11-02

**Authors:** Stephen CW Sze, Ho-Pan Cheung, Tzi-Bun Ng, Zhang-Jing Zhang, Kam-Lok Wong, Hei-Kiu Wong, Yong-Mei Hu, Christine MN Yow, Yao Tong

**Affiliations:** 1 School of Chinese Medicine, Li Ka Shing Faculty of Medicine, The University of Hong Kong, Pokfulam, Hong Kong SAR, China; 2 School of Biomedical Sciences, Faculty of Medicine, The Chinese University of Hong Kong, Shatin, N.T., Hong Kong SAR, China; 3 Medical Laboratory Science Section, Department of Health Technology and Informatics, Hong Kong Polytechnic University, Hung Hom, Hong Kong SAR, China

## Abstract

**Background:**

The prevalence and risk of cardiovascular disease increase after menopause in correlation with the progression of abnormality in the serum lipid profile and the deprivation of estrogen. *Erxian *decoction (EXD), a Chinese medicinal formulation for treating menopausal syndrome, stimulates ovarian estrogen biosynthesis. This study investigates whether EXD improves the serum lipid profile in a menopausal rat model.

**Methods:**

Twenty-month-old female Sprague Dawley rats were treated with EXD and its constituent fractions. Premarin was administered for comparison. After eight weeks of treatment, rats were sacrificed and the serum levels of total cholesterol, triglyceride, high-density-lipoprotein cholesterol and low-density-lipoprotein cholesterol were determined. The hepatic protein levels of 3-hydroxy-3-methyl-glutaryl-CoA reductase and low-density-lipoprotein receptor were assessed with Western blot.

**Results:**

The serum levels of total cholesterol and low-density-lipoprotein cholesterol were significantly lower in the EXD-treated group than in the constituent fractions of EXD or premarin groups. However, the serum levels of triglyceride and high-density-lipoprotein cholesterol were not significantly different from the control groups. Results from Western blot suggest that EXD significantly down-regulated the protein level of 3-hydroxy-3-methyl-glutaryl-CoA reductase and up-regulated low-density-lipoprotein receptor. **Conclusion **EXD improves serum lipid profile in a menopausal rat model through the suppression of the serum levels of total cholesterol and low-density-lipoprotein cholesterol, possibly through the down-regulation of the 3-hydroxy-3-methyl-glutaryl-CoA and up-regulation of the low-density-lipoprotein receptor.

## Background

The risk of cardiovascular disease (CVD) and the mortality due to CVD are higher in menopausal women than their premenopausal counterparts [1,2]. The blood lipid profile of women deteriorates after menopause. Derby *et al*. demonstrated that the serum levels of total cholesterol (TC), low-density-lipoprotein cholesterol (LDL-C) and triglyceride (TG) peak at a later stage of menopause [3]. A sex difference in the expression of cholesterol regulating enzymes was also found in an animal model [4], suggesting that the adverse lipid profile may be related to estrogen deficiency. Administration of female hormones may produce favorable effects on the serum lipid profile in postmenopausal women [5].

*Erxian *decoction (EXD), consisting of *Curculigo orchioides *(*Xianmao*), *Epimedium brevicornum *(*Yinyanghuo*), *Morinda officinalis *(*Bajitian*), *Angelica sinensis *(*Danggui*), *Phellodendron chinense *(*Huangbo*) and *Anemarrhena asphodeloides *(*Zhimu*), has long been used in Chinese medicine to relieve menopausal symptoms [6]. Our previous study demonstrated that EXD effectively induces ovarian production of estradiol *via *the up-regulation of ovarian aromatase, a key enzyme in the biosynthesis of estradiol [6]. The antihyperlipidemic properties exhibited by some bioactive compounds of EXD further suggest the possible effect of EXD on improving the blood lipid profile. Ferulic acid from *Angelica sinensis *was found to slightly lower liver and plasma TC levels in male Sprague Dawley (SD) rats [7]. Mangiferin from *Epimedium brevicornum*, on the other hand, significantly decreased serum TC, TG and LDL-C and increased HDL-C level in an diabetic rat model [8]. Extract of *Phellodendron chinense *containing various alkaloids such as berberine, palmatine and jatrorrhizine reduced the serum levels of TC and LDL-D in a hypercholesterolemic Wistar rat model [9]. Based on the estrogenic effect of EXD and the hypolipidemic effects of its constituent compounds, we would like to know whether EXD is able to improve the lipid profile in an well-established menopausal rat model [10].

This study investigates whether EXD improves the serum lipid profile in a menopausal rat model and the possible mechanisms involved.

## Methods

### Herbal materials

EXD was prepared according to our previous study [6]. Petroleum, ethyl acetate, *n*-butanol, and methanol, of analytical grade, were purchased from Merck (Germany). HPLC-grade acetonitrile for HPLC analysis was purchased from Merck (Germany). Double distilled water was filtered though a 0.22 μm nitrocellulose membrane (Mili Q, USA).

### Preparation of EA, BU, WA fractions of EXD and their quality analysis

Dried EXD powder (1124 g) was decocted in 1.1L of distilled water at 60°C for 30 minutes and filtered to remove the insoluble plant residues. The filtrate was defatted with petroleum ether and then partitioned with ethyl acetate (EA) and n-butanol (BU) respectively, to produce three different fractions. The ethyl acetate fraction (EA, 26 g) contained mainly fatty acids, aromatic acids, quinoids and sesquiterpene according to an HPLC analysis. The butanol fraction (BU, 109 g) contained mainly flavonol glycosides, saponin glycosides and berberine-type alkaloids, whereas the remaining aqueous fraction (WA, 542 g and POLY, 280 g) which accounted for much of the total EXD weight, contained predominantly polysaccharides (POLY), monosaccharides, inorganic salt and amino acids.

For evaluation of quality consistency among EXD extracts and its constituent fractions, three batches (0.5 g powder each) of EXD and its constituent fractions (BU, EA and WA) were weighed separately and extracted with 10 ml of 75% methanol in a water bath at 60°C for 15 minutes, followed by ultrasonication for 30 minutes. Centrifugation of the extracted mixture at 17,700× *g *(Beckman Coulter, USA) was performed for 15 minutes and the supernatant was saved. The supernatant was filtered through a 0.45 μm Millex^® ^Syringe filter unit, and then injected in a volume of 10 μl for HPLC. Seven standard chemicals including mangiferin, ferulic acid, icariin, curculigoside, jatrorrhizine, palmatine and berberine, which are well-known compounds in EXD, were employed and compared to the fingerprint of EXD. A reverse-phase column XBridge™ C_18 _(4.6 mm×250 mm, 5.0 μm, NO. 186003117, Thermo, USA) connected with a guard column (4.6 mm×7.5, 5.0 μm) was used. The mobile phase consisted of acetonitrile (solvent A) and 0.05% sodium dodecyl sulfate (SDS) in 0.1% acetic acid (solvent B) with a gradient program of 5-18% solvent A in 0-20 minutes, 18%-30% solvent A in 20-30 minutes, 30% solvent A in 30-40 minutes, 30-55% solvent A in 40-60 minutes, 55-65% solvent A in 60-65 minutes, 65-85% solvent A in 65-80 minutes. The flow rate was 1.0 ml per minute. DAD detector was set at 258, 283 and 345 nm for obtaining chromatograms with the maximum number of peaks. UV spectra were obtained from 200 to 400 nm. Chromatogram and peak integration were analyzed with Waters Millennium 32 Chromatography Manager Version 3.3 (Waters, USA).

### Animals

Twenty-month-old female SD rats with a low serum estradiol level were employed as a study model. The animals were purchased at the age of eight months from Animal Laboratory Units, The University of Hong Kong. The animals were housed in an air-conditioned room at an ambient temperature of 24°C and a relative humidity of 50-65% with automatic 12-hour light-dark cycles. The experiment was approved by the Committee on the Use of Live Animals in Teaching and Research (CULATR) of the Li Ka Shing Faculty of Medicine, The University of Hong Kong.

### Drug administration, serum and organ harvesting

Rats were arbitrarily divided into six groups of 36 animals. The Chinese medicine extracts were dissolved in water and fed to the rats through a feeding tube for eight weeks (EXD: 4.10 g/kg; EA: 0.11 g/kg; BU: 0.470 g/kg; WA: 2.34 g/kg) in a volume of approximately 1 ml. Premarin (PRE, an equine estrogen; 31.25 mg/kg; 0.3 mg of estrogen per capsule) was used as the western medication group. This dosage was calculated based on the FDA's guideline of conversion of animal dose to human equivalent dose. Body weights of the rats were measured for dose calculation and drug content was mixed well to ensure uniform intake. The control received the same volume of water as EXD. At the end of the experiments, the animals were anesthetized by an intraperitoneal injection of ketamine (80 mg/kg) and xylazine (10 mg/kg) dissolved in 0.9% saline. The sera and livers were collected and stored at -80°C until experiments.

### Detection of serum lipid level

The serum lipid level was measured with commercially available kits from Stanbio Laboratory (USA). The serum TC, TG, HDL-C and LDL-C levels were measured with the Stanbio Cholesterol LiquiColor^® ^kit, Stanbio Triglyceride LiquiColor^® ^kit, Direct HDL-Cholesterol LiquiColor^® ^and Direct LDL-Cholesterol LiquiColor^® ^kit respectively, according to the manufacturer's instructions.

### Immunoblotting analysis of hepatic enzymes

Liver tissues were ground in liquid nitrogen into a fine powder. Protein was then extracted with RIPA lysis buffer (Sigma-Aldrich, USA) containing a protease inhibitor mix (GE Healthcare, UK). The mixture was centrifuged at 15,700× *g *(Eppendorf, Germany) at 4°C and the supernatant was retained. The protein concentration was quantified by the Bradford assay (Bio-Rad, USA). Protein (120 μg) from each sample was used in SDS-PAGE and the protein was transferred to a PVDF membrane. The hepatic proteins were probed with anti-LDL-receptor (ab30532) (Abcam, Hong Kong) and anti-HMG CoA reductase (sc-27578) (Santa Cruz Biotechnology, USA) antibodies, with the use of anti-GAPDH antibody (MAB374) (Millipore, USA) as a housekeeping gene. The membrane was washed with Tris-buffered saline-Tween 20 (TBS-T) three times, and then incubated with horseradish peroxidase-conjugated (HRP) secondary antibodies (Millipore, USA) in TBS-T buffer for one hour. The chemiluminescence signal was produced with an Amersham ECL™ Advance Western Blotting Detection Kit (GE Healthcare, UK) and detected by a ChemiDoc EQ (Bio-Rad, USA) system.

### Statistical analysis

To evaluate the effect of EXD on serum lipid profile, we compared the mean serum lipid level of the treatment group with the control group by unpaired *t-test*, with Welch's correction for comparison with significantly different variances. Groups treated with EXD's fractions were included to demonstrate the contribution of each fraction to the whole effect of EXD. Differences with *P < 0.05 *were considered as statistically significant. The protein level of the hepatic enzymes was normalized by comparison with GAPDH, the housekeeping enzyme. The relative band intensity in Western blotting of the treatment group was compared with the control group by unpaired *t-test*, with Welch's correction for comparison with significantly different variances and *P < 0.05 *was considered significant. Statistical analysis was conducted with GraphPad Prism 4^® ^(GraphPad Software, USA).

## Results

### Quality control

**Figure 1:**
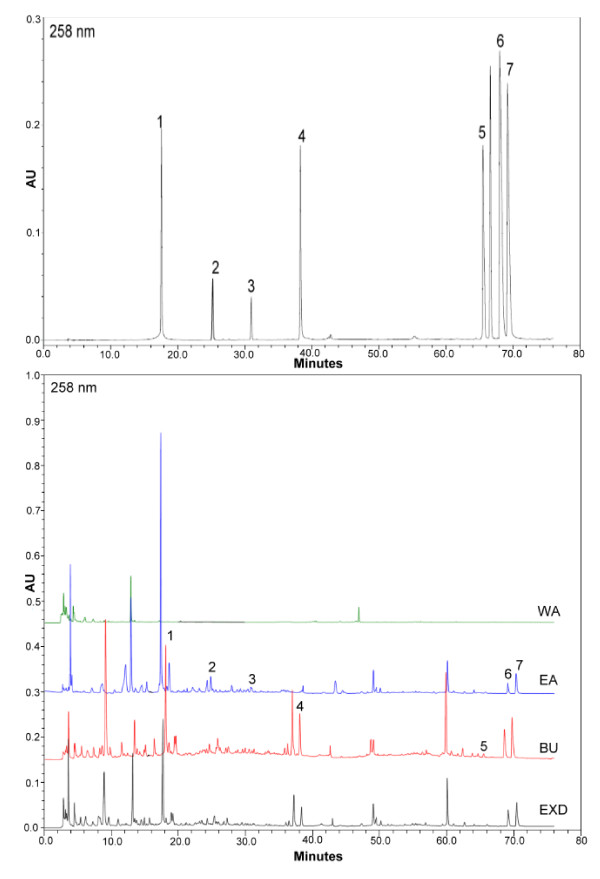
**Comparative chromatograms of EXD and its three different fractions (EA, BU, WA) with seven standard compounds at different UV detection wavelengths (258 nm) 1. Mangiferin; 2. Ferulic acid; 3. Curculigoside; 4. Icariin; 5. Jatrorrhizine; 6. Palmatine; 7. Berberine**.

**Figure 2:**
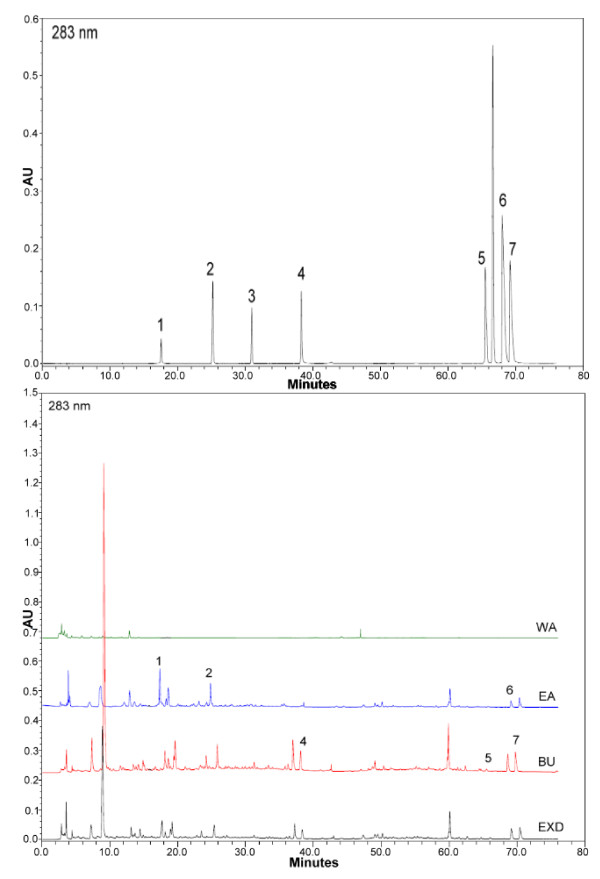
**Comparative chromatograms of EXD and its three different fractions (EA, BU, WA) with seven standard compounds at different UV detection wavelengths (283 nm) 1. Mangiferin; 2. Ferulic acid; 3. Curculigoside; 4. Icariin; 5. Jatrorrhizine; 6. Palmatine; 7. Berberine**.

**Figure 3:**
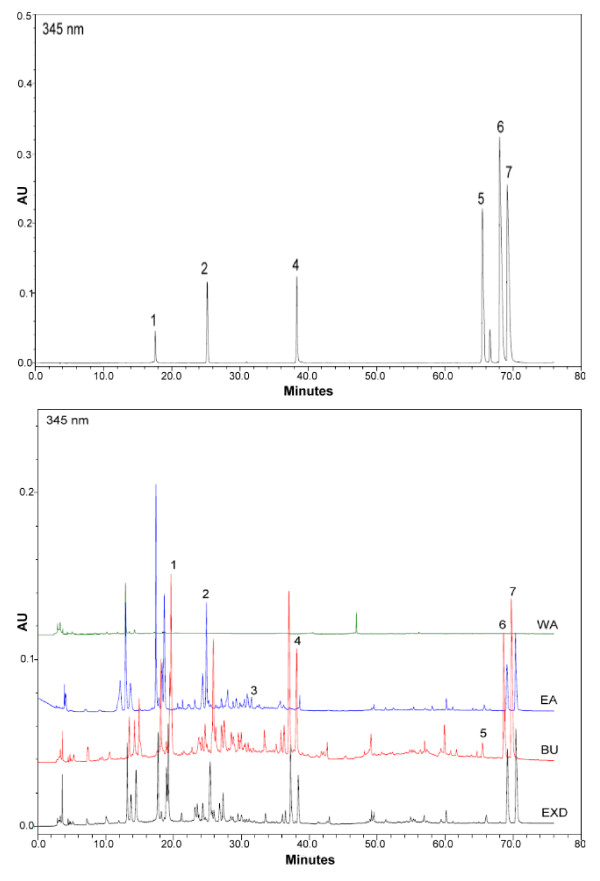
**Comparative chromatograms of EXD and its three different fractions (EA, BU, WA) with seven standard compounds at different UV detection wavelengths (345 nm) 1. Mangiferin; 2. Ferulic acid; 3. Curculigoside; 4. Icariin; 5. Jatrorrhizine; 6. Palmatine; 7. Berberine**.

To explore most of the detectable peaks in the HPLC chromatogram, we investigated the spectra of all eluted peaks found in the chromatogram of EXD and its fractions using photodiode array detection. The chromatograms were generated under the detection wavelengths of 258, 283 and 345 nm. Chromatographic fingerprint showing the elution peaks of seven standard compounds and other common peaks are shown in Figure 1, 2 and 3. Three batches of EXD and its constituent fractions were examined by HPLC under optimal running conditions. The results are shown in Tables 1, 2 and 3. Inter-assay relative standard deviation (RSD) values were less than 5% for all standards. The WA fraction was not included in the tables as the standard compounds were not detected.

**Table 1:** Quantification based on chromatograms of three batches of EXD powders (*n *= 3)

Batch no	Mangiferin	Ferulic acid	Curculigoside	Icariin	Jatrorrhizine	Palmatine	Berberine
S1	3.02	0.62	0.45	0.58	0.02	0.64	1.52
S2	3.02	0.63	0.45	0.56	0.02	0.59	1.49
S3	3.00	0.67	0.45	0.53	0.20	0.64	1.47
Mean	3.01	0.64	0.45	0.56	0.02	0.62	1.49
RSD (%)	1.15	2.60	0.36	2.52	0.06	2.77	2.75

The content of compound in EXD powder (μg/mg)

**Table 2:** Quantification based on chromatograms of three batches of EA powders (*n *= 3)

Batch no	Mangiferin	Ferulic acid	Curculigoside	Icariin	Jatrorrhizine	Palmatine	Berberine
S1	9.19	0.62	0	0	0.01	0.24	0.76
S2	9.13	0.63	0	0	0.02	0.21	0.69
S3	9.16	0.67	0	0	0.11	0.28	0.72
mean	9.16	0.64	0	0	0.01	0.24	0.72
RSD (%)	3.00	3.14			0.25	3.51	3.51

The content of compound in EA powder (μg/mg)

**Table 3:** Quantification based on chromatograms of three batches of BU powders

Batch no	Mangiferin	Ferulic acid	Curculigoside	Icariin	Jatrorrhizine	Palmatine	Berberine
S1	8.18	1.10	0.21	3.12	0.09	2.56	4.78
S2	8.19	0.91	0.26	3.12	0.09	2.49	4.77
S3	8.19	1.05	0.25	3.02	0.09	2.44	4.78
mean	8.19	1.02	0.24	3.09	0.09	2.50	4.78
RSD (%)	0.47	2.40	2.67	5.46	0.08	0.06	0.50

The content of compound in BU powder (μg/mg)

### Effects on lipid profile after EXD treatment

**Figure 4:**
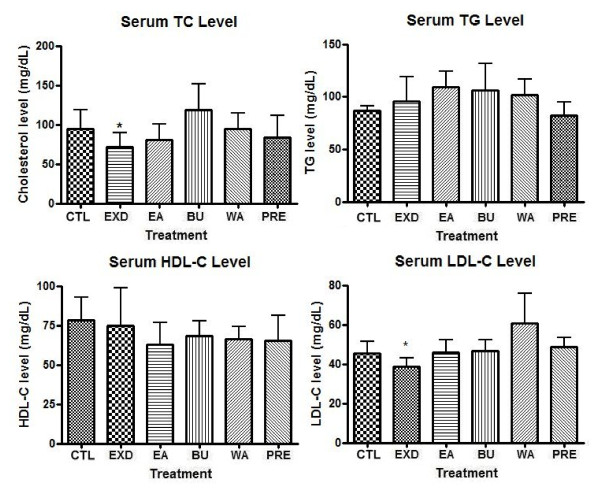
**Serum concentrations of total cholesterol (TC), triglyceride (TG), high density lipoprotein cholesterol (HDL-C) and low density lipoprotein cholesterol (LDL-C) after treatment of old female rats with EXD, its constituent fractions (EA, BU and WA) and premarin (PRE)**. The serum concentrations of TC, TG, HDL-C and LDL-C after EXD treatment; Data are presented in mean ± standard deviation (SD), n ≥ 3. The treatment denoted with * represents *P < 0.05 *in unpaired t-test compared with control group. Welch's correction was made for comparison with significantly different variances. Serum TC level: CTL vs EXD *P *= 0.0231, *t *= 2.204. Serum LDL-C level: CTL vs EXD *P *= 0.0473, *t *= 1.984

Figure 4 shows that the serum TC level in the menopausal SD rat model decreased significantly after oral administration of EXD, while the three fractions of EXD, namely EA, BU and WA, showed no significant effects. The *P *value of the EXD group compared with the control group was less than 0.05 (*P = *0.0231, *t = *2.204) in 95% confidence interval (CI). A slight albeit not statistically significant increase of TC level was observed in the EA, BU and WA groups. Neither was there a significant decrease of the serum TC level in the positive control group fed with premarin.

The effects of EXD and its fractions on serum TG level were not prominent. The serum TG level in all treatment groups showed no significant changes. The serum TG levels in the control, EXD and premarin groups were comparable with each other and slightly higher than those of EA, BU and WA groups.

Similar to the serum level of TG, all treatment groups exhibited no statistically significant effects on the serum level of HDL-C. While the serum HDL-C after EXD treatment was comparable to the control group, the HDL-C levels in those of the EA, BU, WA and premarin groups decreased, although the change was not statistically significant.

However, oral administration of EXD could bring about a significant reduction in the serum LDL-C level. The *P *value of the EXD group was smaller than 0.05 (*P = *0.0473, *t = *1.984) when analyzed by unpaired *t-test *in comparison with the control group. The fractions of EXD, on the other hand, did not elicit significant changes in the serum LDL-C level. The premarin treatment was also unable to suppress the serum LDL-C level.

### Effects on protein level of HMG CoA reductase (HMGCR) and LDL receptor (LDLR) after EXD treatment

**Figure 5:**
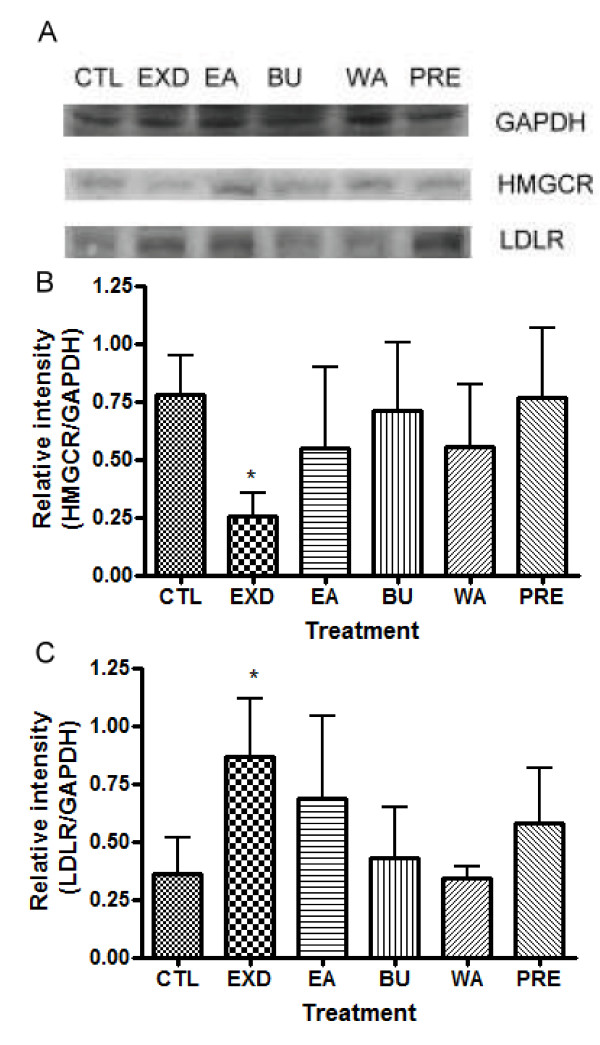
**Relative intensities of HMG-CoA reductase (HMGCR) and low density lipoprotein receptor (LDLR) (normalized with GAPDH in Western blot experiment) after treatment of old female rats with EXD, its constituent fractions (EA, BU and WA) and premarin (PRE)**. The data are presented as mean ± standard deviation (SD), n ≥ 3. The treatment denoted with * represents *P < 0.05 *in unpaired t-test compared with control group. Welch's correction was made for comparison with significantly different variances. HMGCR: CTL vs EXD *P *= 0.0104, *t *= 4.556. LDLR: CTL vs EXD *P = *0.0308, *t *= 2.979

To elucidate the underlying mechanism of the effects of EXD on serum lipid profile, we examined the protein levels of HMG CoA reductase and LDLR using Western blot. The results are summarized in Figure 5.

EXD significantly suppressed the protein level of HMGCR (*P *= 0.0104, *t *= 4.556). The EA, BU and WA fractions of EXD did not significantly decrease the protein level of HMGCR in comparison with the control. In the premarin-treated group, the protein level of HMGCR was comparable to that of the control group. EXD significantly increased the protein level of LDLR (*P = *0.0308, *t *= 2.979), while the EA and PRE treated groups demonstrated a slight albeit insignificant increase. Protein levels of LDLR in the WA and BU groups were comparable to that of the control group.

## Discussion

The risk of cardiovascular disease in older women is one of the major concerns in menopausal health management. It has been reported that about 42% of women older than 65 years of age exhibit a substantial increase in serum level of TC, LDL-C [11] and TG [3]. In this study, we investigated the effects of EXD on serum lipid profiles and its underlying mechanism. HPLC was used to ensure that there were no variations in pharmacological effects of EXD due to sample preparation.

### Sample consistency analysis of EXD and its fractions

The comprehensive HPLC chromatograms of EXD and its fractions were generated and compared with the peaks of seven standards, mangiferin, ferulic acid, icariine, curculigoside, jatrorrhizine, palmatine and berberine (Figure 1, 2 and 3). These chromatographic fingerprints with seven marker compounds were used as reference standards, indicating the purity, identity and quality consistency among EXD and its fractions. The relative standard deviations of the amount of the five standards were less than 5% in three batches of EXD and their constituent fractions (Tables 1, 2 and 3), indicating quality consistency among the different batches of EXD and constituent fractions as well as excluding the influence of any unknown variability or instability found in the composition of the active constituents in the pharmacological test of EXD and its fractions.

The BU fraction (Figure 1, 2 and 3, Table 3) contained most of the marker chemicals (including fenolic acids, flavonoids and alkaloids). The relative contents of the seven marker compounds were much higher compared with EXD (original formula) (Table 1), while the EA fraction (Table 2) showed a much higher content of the small phenols, such as mangiferine and ferulic acid. Most of the compounds were detected at 283 nm. The alkaloids (such as berberine, jatrorrhizine and palmatine) displayed a higher absorption at 345 nm, while phenolic acid (such as mangiferin and ferulic acid) showed ideal absorption at 258 nm. The chromatogram of water fraction (WA) at three different UV detection wavelengths indicated that almost all of the compounds could be completely extracted with ethyl acetate and *n*-butanol, and the residual chemicals in the WA fraction were presumably some small-molecular-weight metabolites, such as sugars, amino acids, organic acids and other trace elements.

### Effects of EXD and its fractions on serum lipid profile

The correlation of low estrogen level with serum lipid profile [11] as well as the stimulatory effect of EXD on ovarian estrogen synthesis[6] suggest that EXD may prevent adverse changes in the lipid profile of a menopausal rat model. The serum TC level in the EXD group was significantly reduced after treatment. The constituent fractions of EXD were less effective in lowering the serum TC level. Thus, we suggest that the hypocholesterolemic effect of EXD is exerted by the complete formulation of EXD rather than any constituent compounds. In the premarin-treated group (human equivalent dosage), the TC level was decreased by an insignificant degree.

On the other hand, this study found that the effects of EXD on the serum TG level were insignificant. In all groups treated with EXD and its constituent fractions, TG level was slightly increased. Interestingly, the increase after EXD treatment was less pronounced compared with its constituent fractions. While EXD did not improve the serum TG level, it did not lead to an increase of TG as its bioactive fractions did. In the premarin-treated group, the TG level was comparable to the control group. The effect of premarin on the serum TG level was similar with some other studies of conjugated estrogen on postmenopausal women, in which conjugated estrogen alone may increase the circulating TG level [5,12,13].

The effect of EXD on the serum HDL-C level was not obvious. The serum HDL-C in all groups treated with EXD or its fractions did not change significantly, while in the groups treated with the constituent fractions the serum HDL-C level decreased slightly, thereby worsening the lipid profile. While EXD cannot improve the serum HDL-C level, it can prevent the lipid profile from worsening in comparison with its constituent fractions. The serum HDL-C level in the premarin-treated group also decreased slightly.

However, EXD could improve the serum lipid profile in our study model by decreasing the serum LDL-C level. As an increased LDL-C level is a risk factor for CVD, our results suggested that EXD may be able to ameliorate the CVD risk in the menopausal model. Again, effects were observed only in the EXD-treated group, while the individual constituent fractions did not significantly reduce the serum LDL-C level. However, similar to the results on the serum HDL-C, oral administration of premarin did not improve the serum lipid profile. The serum LDL-C level in the premarin-treated group was comparable to the control group and no beneficial effects were noted.

Our results on the serum lipid level showed that EXD may act as a preventive medicine for reducing CVD risk in the rat menopausal model by reducing serum TC level and LDL-C level while preventing the HDL-C and TG levels from worsening. However, treatment with premarin did not manifest significant improvement on the serum lipid profile in the rat model. According to studies about hormone replacement therapy on menopausal women, supplements with estrogen could increase the serum HDL-C level and reduce LDL-C level thus reducing the CVD risk factors [5,12]. One possible reason is that, the laboratory animals used in this study were kept in a cage and remained physically inactive. Physical inactivity is suspected as one of the risk factors for adverse lipid profile [14]. Therefore, in the case of this study the hypolipidemic effect of premarin was possibly counteracted. Also, due to different metabolism between rats and human, the metabolism of estrone and equilin in premarin may not be the same in rats and human. Further investigation is needed before it can be confirmed.

The improvement of lipid profile in the EXD-treated group but not in the premarin-treated group raises a question of whether the positive action of EXD is due to its estrogenic effect or is estrogen-independent. Our previous study showed that EXD increased the circulating estrogen level by stimulating ovarian biosynthesis [6]; however, this study did not indicate whether this estrogenic effect improved the lipid profile due to the negative response in the premarin-treated group. In future studies, 17-beta-estradiol may be used as a positive control to facilitate comparison. Also, an ovariectomized rat model may be included to for further elucidation of the possible role of stimulated endogenous estrogen production by EXD.

### Regulation of HMGCR and LDL-R by EXD at translational levels

In order to elucidate the underlying mechanism of the significant improvement in serum profiles of TC and LDL-C demonstrated by EXD, we examined the protein levels of HMGCR and LDLR using Western blot. The levels of HMGCR and LDLR are crucial in maintaining the TC and LDL-C balance. HMGCR is the rate limiting enzyme that controls *de novo *synthesis of cholesterol through mevalonate pathway [15]. HMGCR catalyses the conversion of HMG-CoA into mevalonate, ultimately leading to cholesterol synthesis [16]. LDLR maintains cholesterol homeostasis by a receptor-recycling pathway, in which circulating LDL-C is returned into the liver by receptor-mediated endocytosis [16-18].

Only EXD-treated rats displayed a significantly decreased protein level of HMGCR, indicating a decrease in the *de novo *synthesis of TC. The HMGCR protein levels in the groups treated with other constituent fractions were comparable to each other and showed no significant differences from the control. The protein level of HMGCR in the premarin-treated group was also comparable to those of the control groups. This is consistent with the results of the serum TC level, which did not change significantly in the premarin group. In fact, a previous study suggested that estradiol regulates HMGCR through the activation of AMP-activated protein kinase which phosphorylates HMGCR and inactivates its catalytic property [19] rather than acting at the transcription level.

As the HMGCR level in the EXD-treated group decreased, it is anticipated that the LDLR level would rise. From the results, the increase in LDLR protein level in the EXD-treated group would lead to an elevated clearance of LDL-C, which is in line with the decreased serum LDL-C level. The effect of the constituent fractions of EXD was not as pronounced as that of EXD, suggesting the combined effect of the component fractions in EXD. The increase of protein level of LDL-R in the premarin-treated group was not significant, as expected from the results on serum lipid level and HMGCR level.

Although EXD treatment can significantly suppress the protein level of HMGCR and elevate LDLR level, the effect of EXD on the serum TC and LDL-C levels was not as prominent as that on the relevant regulatory proteins. Such discrepancy may be attributed to the complex regulation of HMGCR and LDLR activity. Moreover, the expression level of HMGCR, the regulation of HMGCR activity involves mechanism such as phosphorylation/dephsphorylation by AMP-dependent kinase (AMPK)/protein phosphatase 2A (PP2A); transcriptional regulation of sterol regulatory element-binding proteins (SREBPs) or protein degradation [15,20]. Pallottini *et al*. revealed that while the protein level of HMGCR remained unchanged, its degradation rate reduced and the enzyme was fully active in aged rats [15]. Whether the discrepancy between the protein level and serum lipid profile is related to the above factors was not assessed in this study.

Our results demonstrated that EXD improved the serum lipid profile in a rat menopausal model through the modulation of the protein levels of HMGCR and LDLR, thus lowering serum TC and LDL-C. Further studies on the synergistic effects of EXD on serum lipid profile and more detailed studies on the mechanisms of HMGCR and LDLR regulation by EXD will be implemented by us in the near future.

## Conclusion

EXD improves serum lipid profile in a menopausal rat model through the suppression of the serum levels of total cholesterol and low-density-lipoprotein cholesterol, possibly through the down-regulation of the HMGCR and up-regulation of the LDLR.

## Abbreviations

BU: butanol fraction; CVD: cardiovascular disease; EA: ethyl-acetate fraction; EXD: Erxian decoction; HL: hepatic lipase; HDL-C: high density lipoprotein cholesterol; HMGCR: 3-hydroxy-3-methyl-glutaryl-CoA reductase; LDL-C: low density lipoprotein cholesterol; LDLR: low density lipoprotein receptor; TC: total cholesterol; TG: triglyceride; SD rat: Sprague-Dawley rat; WA: aqueous fraction; TBS-T: Tris-buffered saline-Tween 20

## Competing interests

The authors declare that they have no competing interests.

## Authors' contributions

SCWS designed the study, assisted in the animal experiments, and wrote the manuscript. HPC conducted the experiments on the serum lipid profile and Western blotting and assisted in the writing of the manuscript. TBN and ZJZ designed the experiments and analyzed the data. KLW conducted the animal experiments. HKW and YMH analyzed the extraction fractions and conducted quality control experiments. CMNY assisted in the design of the experiments on serum lipid profile. YT coordinated the study and amended the manuscript. All authors read and approved the final manuscript.

## References

[B1] Kolovou GD Bilianou HG Influence of aging and menopause on lipids and lipoproteins in women Angiology 2008 59 54s 57s 10.1177/0003319708319645 18515273

[B2] Agrinier N Cournot M Dallongeville J Arveiler D Ducimetiere P Ruidavets JB Ferrieres J Menopause and modifiable coronary heart disease risk factors: A population based study Maturitas 2010 65 237 243 10.1016/j.maturitas.2009.11.023 20031345

[B3] Derby CA Crawford SL Pasternak RC Sowers M Sternfeld B Matthews KA Lipid Changes During the Menopause Transition in Relation to Age and Weight Am J Epidemiology 2009 169 1352 1361 10.1093/aje/kwp043 PMC2727246 19357323

[B4] De Marinis E Martini C Trentalance A Pallottini V Sex differences in hepatic regulation of cholesterol homeostasis J Endocrinology 2008 198 635 643 10.1677/JOE-08-0242 18603607

[B5] Vaziri SM Evans JC Larson MG Wilson PWF The Impact of Female Hormone Usage on the Lipid Profile - the Framingham Offspring Study Arch Intern Med 1993 153 2200 2206 8215723

[B6] Sze SC Tong Y Zhang YB Zhang ZJ Lau AS Wong HK Tsang KW Ng TB A novel mechanism: Erxian Decoction, a Chinese medicine formula, for relieving menopausal syndrome J Ethnopharmacol 2009 123 27 33 10.1016/j.jep.2009.02.034 19429335

[B7] Kamal-Eldin A Frank J Razdan A Tengblad S Basu S Vessby B Effects of dietary phenolic compounds on tocopherol, cholesterol, and fatty acids in rats Lipids 2000 35 427 435 10.1007/s11745-000-541-y 10858028

[B8] Muruganandan S Srinivasan K Gupta S Gupta PK Lal J Effect of mangiferin on hyperglycemia and atherogenicity in streptozotocin diabetic rats J Ethnopharmacology 2005 97 497 501 10.1016/j.jep.2004.12.010 15740886

[B9] Yuan LJ Tu DW Ye XL Wu JP Hypoglycemic and hypocholesterolemic effects of Coptis chinensis franch inflorescence Plan Food for Hum Nutr 2006 61 139 144 10.1007/s11130-006-0023-7 17031605

[B10] Liao BS Hu Y Ju G Fang ZQ Liao H Zhang BN Erxian tang dui shi ba yue ling cixing dashu xiaqiunao- chuiti- luanchao zhou gongneng de tiaojie Shandong Zhongyi Xueyuan Xuebao 1996 20 396 398

[B11] Dalal D Robbins JA Management of hyperlipidemia in the elderly population: an evidence-based approach South Med J 2002 95 1255 1261 12539990

[B12] Bayrak A Aldemir D Bayrak T Dursun P Corakci A The effect of hormone replacement therapy on the levels of serum lipids, apolipoprotein Al, apolipoprotein B and lipoprotein (A) in Turkish postmenopausal women Febs Journal 2006 273 213 213 10.1007/s00404-006-0187-2 16810536

[B13] Godsland IF Effects of postmenopausal hormone replacement therapy on lipid, lipoprotein, and apolipoprotein (a) concentrations: analysis of studies published from 1974-2000 Fertil Steril 2001 75 898 915 10.1016/s0015-0282(01)01699-5 11334901

[B14] Gulanick M Cofer LA Coronary risk factors: influences on the lipid profile J Cardiovasc Nurs 2000 14 16 28 10.1097/00005082-200001000-00004 10653273

[B15] Pallottini V Montanari L Cavallini G Bergamini E Gori Z Trentalance A Mechanisms underlying the impaired regulation of 3-hydroxy-3-methylglutaryl coenzyme A reductase in aged rat liver Mech Ageing Dev 2004 125 633 639 10.1016/j.mad.2004.08.001 15491682

[B16] Goldstein JL Brown MS Regulation of the mevalonate pathway Nature 1990 343 425 430 10.1038/343425a0 1967820

[B17] Jeon H Blacklow SC Structure and physiologic function of the low-density lipoprotein receptor Annu Rev Biochem 2005 74 535 562 10.1146/annurev.biochem.74.082803.133354 15952897

[B18] Rang HP Dale MM Ritter JM PK M Atherosclerosis and Lipoprotein Metabolism Pharmacology 2003 5 New York: Churchill Livingstone 306 310

[B19] Trapani L Violo F Pallottini V Hypercholesterolemia and 3-hydroxy-3-methylglutaryl coenzyme A reductase regulation in aged female rats Exp Gerontol 2010 45 119 128 10.1016/j.exger.2009.10.014 19895880

[B20] Pallottini V Martini C Cavallini G Donati A Bergamini E Notarnicola M Caruso MG Trentalance A Modified HMG-CoA reductase and LDLr regulation is deeply involved in age-related hypercholesterolemia J Cell Biochem 2006 98 1044 1053 10.1002/jcb.20951 16741953

